# Improving documentation and coding for acute organ dysfunction biases estimates of changing sepsis severity and burden: a retrospective study

**DOI:** 10.1186/s13054-015-1048-9

**Published:** 2015-09-14

**Authors:** Chanu Rhee, Michael V. Murphy, Lingling Li, Richard Platt, Michael Klompas, for the Centers for Disease Control and Prevention Epicenters Program

**Affiliations:** Department of Population Medicine, Harvard Medical School and Harvard Pilgrim Health Care Institute, 133 Brookline Avenue, Boston, MA 02215 USA; Division of Infectious Diseases, Brigham and Women’s Hospital, 75 Francis Street, Boston, MA 02115 USA

## Abstract

**Introduction:**

Claims-based analyses report that the incidence of sepsis-associated organ dysfunction is increasing. We examined whether coding practices for acute organ dysfunction are changing over time and if so, whether this is biasing estimates of rising severe sepsis incidence and severity.

**Methods:**

We assessed trends from 2005 to 2013 in the annual sensitivity and incidence of discharge ICD-9-CM codes for organ dysfunction (shock, respiratory failure, acute kidney failure, acidosis, hepatitis, coagulopathy, and thrombocytopenia) relative to standardized clinical criteria (use of vasopressors/inotropes, mechanical ventilation for ≥2 consecutive days, rise in baseline creatinine, low pH, elevated transaminases or bilirubin, abnormal international normalized ratio or low fibrinogen, and decline in platelets). We studied all adult patients with suspected infection (defined by ≥1 blood culture order) at two US academic hospitals.

**Results:**

Acute organ dysfunction codes were present in 57,273 of 191,695 (29.9 %) hospitalizations with suspected infection, most commonly acute kidney failure (60.2 % of cases) and respiratory failure (28.9 %). The sensitivity of all organ dysfunction codes except thrombocytopenia increased significantly over time. This was most pronounced for acute kidney failure codes, which increased in sensitivity from 59.3 % in 2005 to 87.5 % in 2013 relative to a fixed definition for changes in creatinine (*p* = 0.019 for linear trend). Acute kidney failure codes were increasingly assigned to patients with smaller creatinine changes: the average peak creatinine change associated with a code was 1.99 mg/dL in 2005 versus 1.49 mg/dL in 2013 (*p* <0.001 for linear decline). The mean number of dysfunctional organs in patients with suspected infection increased from 0.32 to 0.59 using discharge codes versus 0.69 to 0.79 using clinical criteria (*p* <0.001 for both trends and comparison of the two trends). The annual incidence of hospitalizations with suspected infection and any dysfunctional organ rose an average of 5.9 % per year (95 % CI 4.3, 7.4 %) using discharge codes versus only 1.1 % (95 % CI 0.1, 2.0 %) using clinical criteria.

**Conclusions:**

Coding for acute organ dysfunction is becoming increasingly sensitive and the clinical threshold to code patients for certain kinds of organ dysfunction is decreasing. This accounts for much of the apparent rise in severe sepsis incidence and severity imputed from claims.

## Introduction

Administrative claims data are used extensively to describe the epidemiology of severe sepsis [1]. Analyses of large claims databases have suggested a dramatic rise in the incidence of severe sepsis and sepsis-associated organ dysfunction over time, helping spur global recognition of its importance [2–6]. Claims data have also suggested declines in sepsis-associated mortality rates [3, 4, 7, 8]. In addition, the United States (US) Centers for Medicare and Medicaid Services (CMS) has recently proposed monitoring hospitals’ adherence to severe sepsis bundles using claims data to screen for eligible patients followed by chart review [9].

Despite the convenience of administrative data, however, their accuracy for tracking changes in sepsis burden over time is controversial [10, 11]. There is evidence that increasing awareness of sepsis among clinicians and hospital coders, coupled with financial incentives to code for higher acuity of illness, is leading clinicians to diagnose and code for sepsis more diligently [12, 13]. In practice, though, most epidemiologic studies of sepsis incidence do not use the explicit *International Classification of Diseases*, *Ninth Revision*, *Clinical Modification* (ICD-9-CM) diagnosis codes for severe sepsis (995.92) and septic shock (785.52) alone, partly because these codes were only introduced in 2002 and partly because chart audits suggest that these codes are still underused [14, 15]. A more common and more sensitive method for estimating the incidence of severe sepsis is to seek patients with concurrent codes for infection and acute organ dysfunction, with or without explicit sepsis codes [16]. It is plausible, however, that the same pressures leading to better coding for sepsis are also leading to more sensitive coding for acute organ dysfunction, which in turn could be biasing estimates of the incidence, severity, and mortality of severe sepsis [4].

Our aim was to examine temporal trends in the incidence and sensitivity of claims codes for acute organ dysfunction relative to objective clinical markers of acute organ dysfunction utilizing an electronic clinical database that spans a 9-year period at two large academic hospitals. We hypothesized that 1) the sensitivity of coding for acute organ dysfunction has increased over time, 2) the clinical thresholds for coding patients for acute organ dysfunction has decreased, and 3) that these two effects are biasing estimates of temporal trends in the incidence, severity, and mortality of severe sepsis.

## Methods

We identified all patients aged ≥18 years admitted to Massachusetts General Hospital (MGH) and Brigham and Women’s Hospital (BWH) in Boston, Massachusetts between January 1, 2005 and December 31, 2013 and who had evidence of suspected infection, defined as any blood culture order during hospitalization. We retrieved patients’ ICD-9-CM codes, demographics, medications, laboratory results, and hospitalization dates from the hospitals' Research Patient Data Registry; all of these data elements have been captured in this clinical database since 2002 [17, 18]. Dates of mechanical ventilation were obtained from clinical data collected by respiratory therapists at each hospital. We derived patients’ comorbidities from their ICD-9-CM and diagnosis-related group (DRG) codes using the method of Elixhauser [19]. The study was approved by the Partners Healthcare Institutional Review Board (protocol number 2012P002136) and a waiver of patient consent was obtained.

### Trends in acute organ dysfunction in patients with suspected infection

We estimated rates of acute organ dysfunction using codes from widely cited claims-based studies of sepsis epidemiology [3, 6, 16]. We focused on codes for organ dysfunction that can be clearly defined using electronic clinical data. Our clinical definitions for organ dysfunction were informed by thresholds suggested by the Surviving Sepsis Campaign Guidelines and the Sepsis-related Organ Failure Assessment score [20, 21], but were modified to incorporate changes in baseline organ function (Table 1). Furthermore, because we wanted these electronic criteria to have high specificity, we chose conservative clinical and laboratory thresholds that would undeniably qualify a patient as having acute organ dysfunction by virtually any definition. We calculated the sensitivity of each set of organ dysfunction codes for clinical markers of organ dysfunction for each calendar year. We also examined whether the threshold for coding for acute organ dysfunction has changed over time by looking for temporal changes in the positive predictive value (PPV) for each set of organ dysfunction codes. In order to estimate the effect of changing organ dysfunction coding practices on apparent severe sepsis trends, we compared the annual incidence and hospital mortality of patients with suspected infection and at least one organ dysfunction code versus those with suspected infection and at least one clinical marker for organ dysfunction.

**Table 1:** Organ dysfunction categories with corresponding ICD-9-CM codes and objective clinical markers

Organ dysfunction	ICD-9-CM codes^a^	Objective clinical marker
Shock	785.5 (Shock)	Any vasopressor or inotrope^b^ during hospitalization
Respiratory	518.81 (Acute respiratory failure)	Mechanical ventilation for ≥2 consecutive days^c^
	518.82 (Other pulmonary insufficiency)	
	799.1 (Respiratory arrest)	
Renal	584 (Acute kidney failure)	Peak creatinine ≥2.0 mg/dL and ≥1.5× increase from baseline^d^
Acidosis	276.2 (Acidosis)	Nadir pH <7.15^e^
Hepatic	570 (Acute and subacute necrosis of liver)	Peak aspartate aminotransferase (AST) or alanine aminotransferase (ALT) during hospitalization >1000 units/L, or total bilirubin ≥6.0 mg/dL
	573.3 (Hepatitis unspecified)	
	573.4 (Hepatic infarction)	
Thrombocytopenia	287.3 (Thrombocytopenia)	Nadir platelet count during hospitalization <50,000/μL and >50 % decrease from baseline^f^
	287.5 (Thrombocytopenia unspecified)	
Coagulopathy	286.6 (Defibrination syndrome)	Nadir fibrinogen during hospitalization <200 mg/dL or peak international normalized ratio (INR) >3.0 and increase by >0.5 from baseline^g^
	286.9 (Other and unspecified coagulation defects)	

*ICD-9-CM International Classification of Diseases, Ninth Revision, Clinical Modification*

^a^Where three- or four-digit codes are listed, all associated subcodes were included

^b^Vasopressor = parenteral order for norepinephrine, dopamine, epinephrine, phenylephrine, or vasopressin. Inotrope = parenteral order for dobutamine or milrinone

^c^Mechanical ventilation required for 2 or more consecutive calendar days, unless death occurs while on mechanical ventilation prior to the 2^nd^ day

^d^Baseline value for creatinine defined as lowest value from 30 days prior to hospital admission through hospital discharge. Excludes patients with end-stage renal disease code (585.6)

^e^Nadir pH could be from arterial or venous blood gas

^f^Baseline value for platelets defined as highest value from 30 days prior to hospital admission through hospital discharge

^g^Baseline value for INR defined as lowest value from 30 days prior to hospital admission through hospital discharge. Excludes any patient with order for warfarin from day 30 from hospitalization admission to hospital discharge

Our denominator for these analyses was any patient with ≥1 blood culture order during hospitalization because this is a key marker of suspected infection that may be less susceptible to changing clinical practice over time than coding for infection or sepsis. However, we conducted a sensitivity analysis using hospitalizations with infection codes as the denominator for all incidence and mortality trends to assess whether observed trends were generalizable to patients outside the blood culture cohort, and to estimate the degree that changing coding practices for acute organ dysfunction might be affecting claims-based estimates of severe sepsis. Our list of infection codes for this sensitivity analysis included the codes for sepsis (995.91), severe sepsis (995.92), septic shock (785.52) and all infection codes used by Dombrovskiy, Martin and Angus et al., for a total of 1280 different codes [3, 6, 16]. We also compared trends in the number of dysfunctional organs measured by codes versus clinical data in patients with blood culture orders and in patients with codes for severe sepsis (995.92).

### Statistical analyses

Nine-year trends were assessed by fitting linear time series models to the observed annual rates. Each model yielded an estimate for the constant annual change in incidence, mortality, sensitivity and/or PPV rates. For estimates of change in incidence, the annual percent change was calculated as the ratio between the fitted annual change and the observed baseline rate in 2005. All analyses were performed using SAS version 9.4 (SAS Institute, Cary, NC, USA). We considered *p* <0.05 to be statistically significant and used two-tailed tests.

## Results

### Patient characteristics and trends in organ dysfunction codes

There were 818,070 adult hospitalizations from 2005 through 2013, of which 191,695 (23.4 %) had suspected infection (i.e., blood culture orders). Of these, 57,273 patients (29.9 %) had a discharge code for acute organ dysfunction. Clinical characteristics of patients with and without organ dysfunction codes are presented in Table 2. Patients with acute organ dysfunction codes tended to be older and had more comorbid illnesses, longer hospital lengths of stay, and higher in-hospital mortality. Among patients with acute organ dysfunction codes, the most common were acute kidney failure (60.2 % of patients) and respiratory failure (28.9 %).

**Table 2:** Baseline characteristics of patients with suspected infection with and without acute organ dysfunction codes (2005–2013)

Patient characteristics	Any organ dysfunction code	No organ dysfunction code
	(n = 57,273)	(n = 134,422)
Median age (interquartile range)	65 (53, 76)	59 (45, 72)
Male sex	32,779 (57.2)	67,041 (49.9)
White race	44,413 (77.6)	102,539 (76.3)
Comorbidities		
Cancer (solid, metastatic, lymphoma)	12,284 (21.5)	31,032 (23.1)
Diabetes (with and without complications)	9947 (17.4)	24,786 (18.4)
Congestive heart failure	13,647 (23.8)	15,352 (11.4)
Liver disease	4484 (7.8)	6896 (5.1)
Lung disease	8374 (14.6)	21,726 (16.2)
Renal disease	10,555 (18.4)	14,350 (10.7)
Acute organ dysfunction codes:		
Hypotension/shock	9948 (17.4)	_
Respiratory	16,552 (28.9)	_
Renal	34,500 (60.2)	_
Acidosis	8315 (14.5)	_
Hepatic	2875 (5.0)	_
Thrombocytopenia	9426 (16.5)	_
Coagulopathy	2878 (5.0)	_
Median number of organ dysfunction codes	1 (1, 2)	_
Objective clinical markers of organ dysfunction:		
Hypotension/shock	24,181 (42.2)	17,887 (13.3)
Respiratory	18,532 (32.4)	7355 (5.5)
Renal	19,094 (33.3)	3352 (2.5)
Acidosis	10,236 (17.9)	2093 (1.6)
Hepatic	6010 (10.5)	2526 (1.9)
Thrombocytopenia	9703 (16.9)	7980 (5.9)
Coagulopathy	5871 (10.3)	3147 (2.3)
Median number of objective dysfunctional organs	1 (0, 2)	0 (0, 0)
Median hospital length of stay (interquartile range)	11 (6, 20)	6 (4, 11)
Hospital mortality	9265 (16.2)	2775 (2.1)

There was a significant linear increase from 2005 to 2013 in the annual rate of acute organ dysfunction codes in hospitalizations with suspected infection for all types of organ dysfunction except coagulopathy (Table 3). There was also an increase in the incidence of each type of organ dysfunction imputed from objective clinical data; however, the increase in organ dysfunction using clinical criteria was less pronounced for all types of organ dysfunction except thrombocytopenia. In the case of renal failure and respiratory failure, the trends were discrepant, as rising rates of acute kidney failure and respiratory failure codes were contrasted by declining rates of corresponding clinical markers.

**Table 3:** Annual incidence of hospitalizations with suspected infection and organ dysfunction (codes or clinical markers) in 2005 versus 2013

	Organ dysfunction codes				Objective clinical markers of organ dysfunction			
	2005	2013	Fitted 9-year relative change (95 % CI)	*p* value for linear trend	2005	2013	Fitted 9-year relative change (95 % CI)	*p* value for linear trend
	Per/10,000 (Total cases)	Per/10,000 (Total cases)			Per/10,000 (Total cases)	Per/10,000 (Total cases)		
Shock	65 (580)	271 (1787)	+229 % (+176, 282 %)	<0.001	465 (4127)	586 (5279)	+29 % (+16, 42 %)	0.007
Respiratory	171 (1516)	248 (2234)	+55 % (44, 66 %)	<0.001	330 (2933)	314 (2831)	−6.9 % (−8.6, −5.1 %)	<0.001
Renal	322 (2857)	547 (4924)	+81 % (+74, 87 %)	<0.001	354 (3144)	260 (2338)	−27 % (−53, −0.5 %)	0.103
Acidosis	54 (479)	151 (1356)	+187 % (+169, 205 %)	<0.001	76 (673)	80 (722)	+0.6 % (−5.2, +6.5 %)	0.837
Hepatic	26 (232)	42 (381)	+65 % (+47, 83 %)	<0.001	90 (797)	107 (962)	+17 % (−1.6, +36 %)	0.133
Coagulopathy	32 (287)	37 (334)	+39 % (+0.1, 77 %)	0.106	116 (1033)	106 (954)	+4.7 % (−8.2, +18 %)	0.507
Thrombocytopenia	106 (940)	150 (1351)	+51 % (+15, 87 %)	0.039	187 (1657)	264 (2382)	+50 % (+37, 64 %)	<0.001

*CI* confidence interval

The mean number of dysfunctional organs in patients with suspected infection increased from 0.32 to 0.59 using discharge codes (fitted 9-year increase of 87 % relative to 2005, 95 % CI 82, 93 %, *p* <0.001 for linear trend). In contrast, the mean number of organ dysfunctions only rose from 0.69 to 0.79 when using clinical criteria (fitted 9-year increase of 14 % relative to 2005, 95 % CI 11, 16 %, *p* <0.001; *p* <0.001 for comparison of the code-based trend versus the clinical data-based trend). When examining patients coded for severe sepsis (995.92), the annual mean number of coded dysfunctional organs increased from 1.78 in 2005 to 2.21 in 2013 (*p* = 0.060 for linear increase). In contrast, there was a significant decline in the mean number of dysfunctional organs measured by clinical criteria, from 2.87 in 2005 to 2.53 in 2013 (*p* = 0.045 for linear decline; *p* <0.001 for comparison of two trends).

### Sensitivity and PPV of organ dysfunction codes

**Fig. 1:**
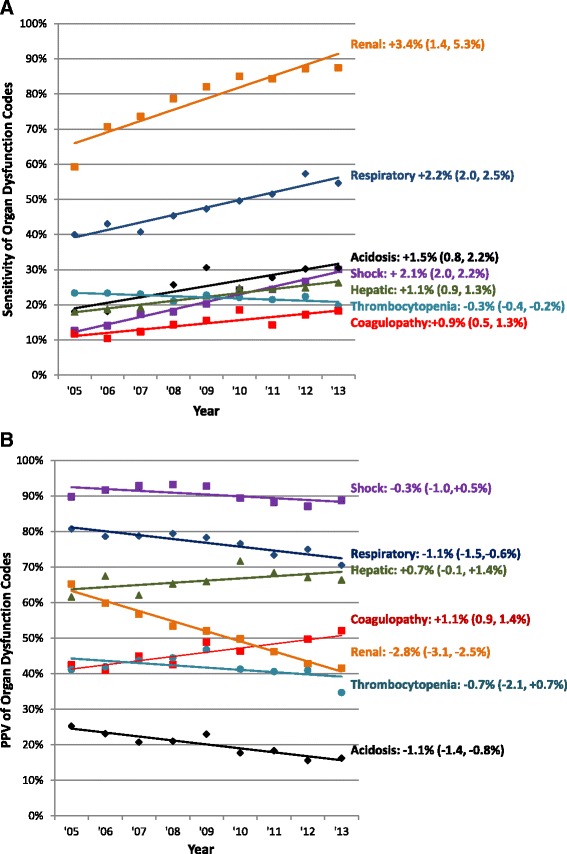
Changing **a** sensitivity and **b** positive predictive value of acute organ dysfunction codes relative to clinical data. Percentages next to organ dysfunction type indicate the fitted annual change in sensitivity relative to 2005, with associated 95 % CIs. *CI* confidence interval

The sensitivity of organ dysfunction coding relative to objective clinical signs of organ dysfunction increased steadily over time for each category, with the sole exception of thrombocytopenia codes (Fig. 1a). This was most pronounced for acute kidney failure codes, which increased in sensitivity from 59.3 % in 2005 to 87.5 % in 2013 (*p* = 0.019 for linear trend), and for respiratory failure codes, which increased from 40.0 % to 54.6 % during the same time period (*p* <0.001).

**Fig. 2:**
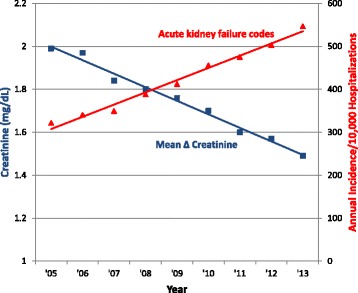
Decreasing mean creatinine change associated with acute kidney failure codes with simultaneous rise in codes. *Blue line* represents the declining annual mean ∆ creatinine (peak – baseline creatinine) associated with an ICD-9-CM code for acute kidney failure (584x) over time. *Red line* represents the rising incidence of hospitalizations with acute kidney failure codes. Excludes patients with codes for end-stage renal disease (585.6). ICD-9-CM, *International Classification of Diseases*, *Ninth Revision*, *Clinical Modification*

Simultaneously, there was a decrease in the PPV of several types of organ types of organ dysfunction codes relative to objective clinical criteria (Fig. 1b). This was again most pronounced for acute kidney failure codes, which decreased in PPV from 65.2 % in 2005 to 41.5 % in 2013 (*p* <0.001 for linear trend). The mean rise in creatinine associated with assigning a discharge code for acute kidney failure decreased from 1.99 mg/dL in 2005 (n = 2088) to 1.49 mg/dL (n = 4804) in 2013 (*p* <0.001 for linear decline) (Fig. 2). The PPV also decreased for respiratory failure codes from 80.8 % to 70.5 % (*p* = 0.005), and a significant decrease from 25.2 % to 16.2 % was also seen for acidosis codes (*p* <0.001). In contrast, there was no significant change in PPV for shock, hepatic, and thrombocytopenia codes, while the PPV for coagulopathy codes increased.

### Incidence and mortality trends

**Fig. 3:**
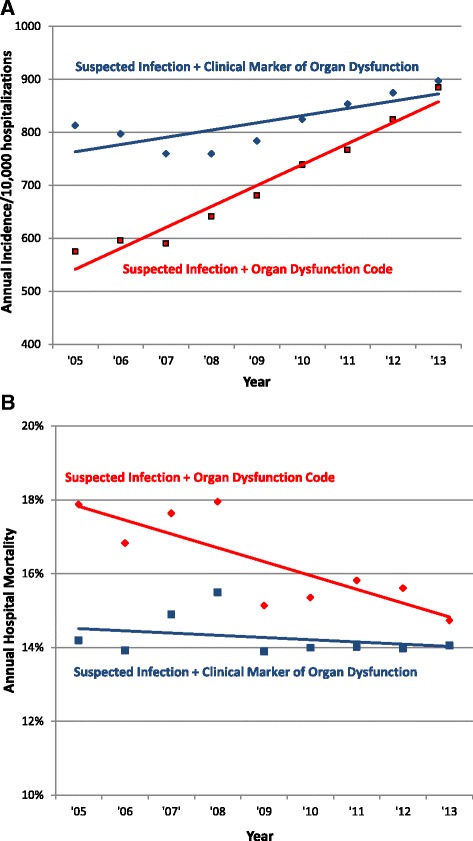
Trends in **a** incidence and **b** mortality with suspected infection and acute organ dysfunction defined by discharge codes versus clinical data. “Suspected infection” defined by the presence of ≥1 blood culture order during hospitalization

There was a significant difference in trends in the incidence and mortality of hospitalizations with acute organ dysfunction when using codes for organ dysfunction versus objective clinical criteria (Fig. 3a, b). The incidence rate of hospitalizations with suspected infection and any organ dysfunction code rose from 575 per 10,000 hospitalizations in 2005 to 885 in 2013 (fitted annual increase of 6.9 %, 95 % CI 5.5, 8.4 %, *p* <0.001 for linear trend). In contrast, hospitalizations with suspected infection and clinical markers of organ dysfunction rose from 896 per 10,000 hospitalizations in 2005 to 952 in 2013 (1.1 % increase/year, 95 % CI 0.1 %, 2.0 %, *p* = 0.075).

The mortality rate of patients with suspected infection and any organ dysfunction code decreased from 17.9 % in 2005 to 14.7 % in 2013 (fitted annual decrease of 0.4 %, 95 % CI 0.3, 0.5 %, *p* = 0.001). In contrast, mortality rates were stable among patients with clinical markers of organ dysfunction, changing from 14.1 % in 2005 to 13.8 % in 2013 (0.1 % decrease/year, 95 % CI 0.0, 0.2 %, *p* = 0.080).

**Fig. 4:**
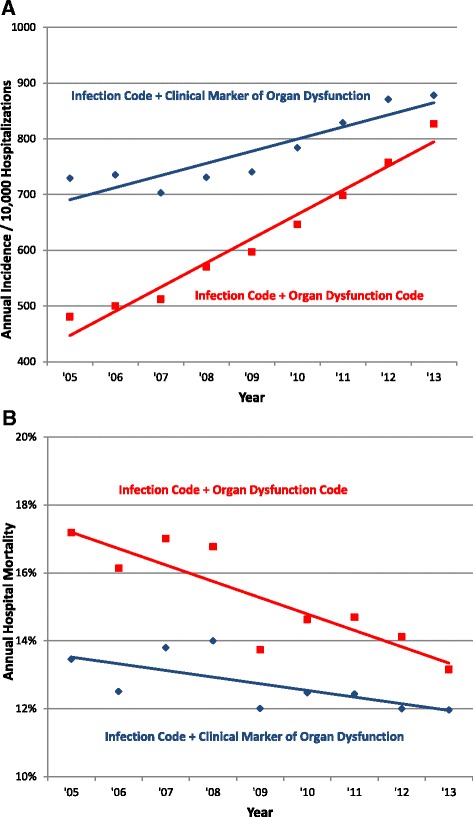
Trends in **a** incidence and **b** mortality with diagnosed infection and acute organ dysfunction defined by discharge codes versus clinical data. “Diagnosed infection” defined by the presence of one of 1280 infection codes at hospital discharge

When this analysis was repeated using patients with an infection discharge code as the denominator, similar trends were found. The incidence of hospitalizations with any infection and organ dysfunction code rose from 481 per 10,000 hospitalizations in 2005 to 827 in 2013 (9.1 %/year, 95 % CI 6.1, 11.7 %, *p* = 0.001) versus a less pronounced rise from 729 to 878 (2.9 %/year, 95 % CI 1.5, 4.3 %, *p* = 0.009) for infection codes plus any clinical marker of organ dysfunction (Fig. 4a). The hospital mortality of any infection and organ dysfunction code decreased from 17.2 % to 13.2 % (0.5 % decline/year, 95 % CI 0.4, 0.6 %, *p* <0.001) versus 13.5 % to 12.0 % (0.2 % decline/year, 95 % CI 0.2, 0.3 %, *p* = 0.002) for infection codes plus any clinical marker of organ dysfunction (Fig. 4b).

## Discussion

In this study, we compared organ dysfunction coding practices to patients’ objective clinical data using 9 years of data from a large electronic clinical database. We found that the sensitivity of hospital discharge codes for identifying hospitalizations with objective signs of acute organ dysfunction increased steadily over time for all organ dysfunction types, with the sole exception of thrombocytopenia. This trend was most striking for patients with acute kidney failure and respiratory failure codes. There was a simultaneous decrease in the positive predictive value for several types of organ dysfunction codes. Most notably, we observed a steady decrease in the average change in creatinine associated with acute kidney failure codes. We also observed a decrease in the proportion of patients assigned respiratory failure codes who required 2 or more days of mechanical ventilation.

Documentation and coding of acute organ failure is known to be imperfect; for example, in one retrospective study, appropriate documentation of acute kidney injury occurred in only 43 % of patients who had a doubling of baseline creatinine [22]. To our knowledge, however, this is the first study to show changes in coding practices relative to objective clinical criteria over an extended period of time. It is likely that coding for organ dysfunction is increasing over time both because of the inherent desire to better document patients’ clinical states, and also because hospitals are eligible for higher reimbursements for caring for more complex patients. Changes in the reimbursement and policy landscape support this trend. For example, in the United States, the Centers for Medicare and Medicaid Services (CMS) transitioned from diagnosis-related group reimbursements into the current Medical Severity DRG (MS-DRG) system in 2007. The MS-DRG system explicitly ties reimbursement to severity of illness and spurred hospitals to make significant efforts to improve documentation and coding [23].

We found that the apparent rate of rise over time in patients with suspected infection and at least one kind of organ dysfunction was markedly higher using claims data compared to objective clinical markers. This suggests that imputing severe sepsis incidence using infection codes and organ dysfunction codes (without necessarily requiring explicit sepsis codes) can be misleading because physicians and hospitals are changing the ways they code for organ dysfunction. The largest contributor to this discrepant increase was a decrease in the threshold for coding for acute kidney failure over time, combined with rising sensitivity for capturing significant changes in baseline creatinine. In addition to financial pressures, the increase in coding for acute kidney injury over time may also be a result of changes in classifications by multidisciplinary collaborative groups that now include smaller changes in baseline serum creatinine [24]. For example, the Acute Kidney Injury Network definition published in 2007 defined a rise in serum creatinine of ≥0.3 mg/dL as the first stage of acute kidney injury; previously, the Risk, Injury, Failure, Loss of kidney function, and End-stage kidney disease (RIFLE) consensus criteria defined a 1.5-fold increase in serum creatinine as the earliest stage of acute kidney injury [25, 26]. Interestingly, thrombocytopenia codes were the only type of organ dysfunction that did not increase in sensitivity in our study. This may be because, in contrast to most of the other types of organ dysfunction, thrombocytopenia is not on CMS’s list of major complications and comorbid conditions that factor most heavily into severity of illness assessment and reimbursements [27].

The mortality decline in patients with suspected infection and objective markers of organ dysfunction was less pronounced than the mortality decrease associated with organ dysfunction codes. This suggests that part of the apparent decline in severe sepsis mortality imputed from claims is likely due to the increasing inclusion of patients with milder organ dysfunction over time. We also found that the increase in mean number of dysfunctional organs was greater when using codes versus clinical data, and in fact the mean number of dysfunctional organs was decreasing in patients coded with severe sepsis (995.92). This indicates that estimating changes in the severity of sepsis based on codes alone is subject to bias, and also supports the notion that the threshold for assigning the explicit severe sepsis code is decreasing. These conclusions are in line with a prior trend analysis of data from the Nationwide Inpatient Sample from 2003 to 2007 that showed a paradoxical increase in the number of coded dysfunctional organ systems in patients with severe sepsis but decreasing in-hospital mortality rates and mean costs per case [4]. A similar phenomenon may account for findings from the National Hospital Discharge Survey that demonstrated an increase in the proportion of patients with sepsis who had any organ failure from 19.1 % in 1979–1984 to 30.2 % in 1995–2000 [6].

Importantly, even in 2013, the sensitivity for most organ dysfunction codes was relatively low (60 % or less in most cases), indicating that claims still substantially underestimate the true occurrence of infection-related organ dysfunction. This suggests that there is still plenty of room for coding accuracy to improve and thus continue to bias future surveillance efforts using claims data. Conversely, if incentives are reversed, it is conceivable that the sensitivity of coding could decrease. A potential example where incentives might change is with the new sepsis bundle mandated by CMS in the US, which proposes to monitor adherence through retrospective review of patients with ICD-10 discharge codes for sepsis, severe sepsis, and septic shock. Measuring changes in any type of disease burden and associated outcomes is centrally dependent on having uniform definitions that are applied consistently over time. Because claims do not live up to this standard in many cases, there is a pressing need to develop objective and efficient surveillance strategies that are more resistant to changes in external forces. The increasing implementation and use of electronic medical record systems worldwide allows for the possibility of shifting surveillance from claims to clinical data, including patients’ laboratory values. These are less prone (although not entirely immune) to changes in use and interpretation over time [10].

Our findings also have implications beyond severe sepsis epidemiology. Several studies unrelated to sepsis have used administrative databases to examine trends in organ dysfunction and also found increasing incidences over time. For example, claims for acute kidney failure in Medicare data rose steadily from 1992 to 2001 while the associated mortality decreased [28]. Likewise, Stefan et al. examined trends in acute respiratory failure using ICD-9-CM codes from the Nationwide Inpatient Sample and found a significant increase in incidence and total costs, but a decline in mortality and length of stay [29].

Our study has several limitations. First, we only used data from two academic hospitals in one city; further studies should explore the generalizability of our findings. Notably, however, our estimated incidence of organ dysfunction and trends in severe sepsis rates as ascertained via ICD-9-CM codes mirror national and international trends [5, 30]. Second, we used blood culture orders as our marker for suspected infection, but it is unclear if this captures the entire cohort of patients with sepsis. However, our findings were identical when using hospitalizations with infection or sepsis diagnoses at discharge, suggesting that these patterns of changing organ dysfunction coding are not unique to patients with blood culture orders. Third, it is possible that some patients being coded as acute respiratory failure are increasingly using noninvasive positive pressure ventilation over time and that therefore we underestimated the sensitivity and overestimated the decline in positive predictive value of claims codes for respiratory failure. However, if this is the case, this also underscores the changing and variable use of the term “respiratory failure” and the need for a more uniform definition. Fourth, we did not evaluate changes in coding for altered mental status since we did not have an objective measure for comparison. Lastly, our estimates of baseline values for laboratory values were derived from the “best” values during or 30 days prior to hospitalization, and this may not be accurate in some cases. However, we applied the same definitions for baseline values over the entire study period, minimizing the risk of any systematic bias.

## Conclusions

In conclusion, the sensitivity of ICD-9-CM coding for clinically defined acute organ dysfunction increased steadily from 2005 through 2013, while the threshold for coding for several types of organ dysfunction decreased. Coding for acute kidney failure, in particular, has increased dramatically. These changes explain a substantial fraction of the reported increase in the incidence of severe sepsis and sepsis-associated organ dysfunction, as well as improvements in survival. Since the coding for these conditions remains incomplete, estimates of the incidence of severe sepsis are likely to continue to increase. There is a pressing need to develop new surveillance strategies for organ dysfunction and sepsis based on clinical data rather than claims codes.

## Key messages

- The sensitivity of coding for acute organ dysfunction is increasing over time.
- Simultaneously, the threshold for coding for several types of organ dysfunction is decreasing, particularly the threshold to code for acute kidney failure.
- These changes explain some of the apparent increase in the incidence of severe sepsis and sepsis-related organ dysfunction, as well as the decline in sepsis-related mortality rates.
- Standardized criteria and surveillance strategies for acute organ dysfunction are needed to enable reliable conclusions to be drawn about trends in the burden of severe sepsis.

## Abbreviations

ALT: alanine aminotransferase
AST: aspartate aminotransferase
BWH: Brigham and Women’s Hospital
CI: confidence interval
CMS: Centers for Medicare and Medicaid Services
ICD-9-CM: *International Classification of Diseases*, *Ninth Revision*, *Clinical Modification*
INR: international normalized ratio
IQR: interquartile range
MGH: Massachusetts General Hospital
MS-DRG: Medical Severity diagnosis-related group
PPV: positive predictive value
US: United States
